# New-Onset Heart Failure and Ischemic Stroke in Non-compaction Cardiomyopathy: A Case Report

**DOI:** 10.7759/cureus.35371

**Published:** 2023-02-23

**Authors:** Harjinder P Singh, Diva Maraj, Elise Hawes, Mumtaz Memon

**Affiliations:** 1 Internal Medicine, Henry Ford Health System, Jackson, USA; 2 Cardiac Imaging, Henry Ford Health System, Jackson, USA; 3 Cardiology, Henry Ford Health System, Jackson, USA

**Keywords:** left ventricular non-compaction, thromboembolism, anticoagulation, heart failure, stroke

## Abstract

Left ventricular non-compaction (LVNC) cardiomyopathy is an embryological disorder of endocardial trabeculation and can cause heart failure, arrhythmias, and thromboembolism. Lifelong anticoagulation is indicated in patients with reduced ejection fraction due to high risks of thromboembolism. Reduced ejection fraction can develop in these patients as a consequence of this cardiomyopathy, thereby increasing the risk of intracardiac thrombus formation. This new-onset reduced ejection fraction may develop rapidly, which may not be amenable to detection by routine screening. We present a case of non-compaction cardiomyopathy (NCC) with a previously normal ejection fraction who had an ischemic stroke and was found to have new-onset reduced ejection fraction.

## Introduction

Left ventricular non-compaction (LVNC) is a rare congenital disorder characterized by a thin, compacted epicardial layer and an extensive non-compacted endocardial layer with prominent trabeculation and deep recesses that communicate with the left ventricular (LV) cavity [1]. The prevalence of LVNC depends on the imaging modality. In previous systematic reviews and meta-analyses, the pooled prevalence in the subset of cardiac patients undergoing cardiac magnetic resonance imaging (MRI) and echocardiography was 14.79% and 1.28%, respectively [2]. Irrespective of age, LVNC can progress to heart failure, arrhythmia, systemic thromboembolism, and sudden cardiac death [3]. Heart failure in LVNC can be caused by systolic or diastolic dysfunction. Systolic dysfunction occurs due to subendocardial hypoperfusion and microcirculatory dysfunction [4], and diastolic dysfunction occurs due to abnormal relaxation and restrictive filling due to multiple prominent trabeculae [5].

Here, we present a patient with known non-compaction cardiomyopathy (NCC) who developed an ischemic stroke likely related to the development of reduced ejection fraction.

## Case presentation

A 61-year-old male with a past medical history of LVNC with no other cardiovascular history or risk factors presented to the emergency room after noticing decreased left-sided peripheral vision for the last two days. He was exercising at that time and did not have any associated headaches, focal weakness, or sensory deficits. He was found to have a left visual field defect suggesting homonymous hemianopia on outpatient visual field evaluation and was asked to go to the emergency room.

**Figure 1:**
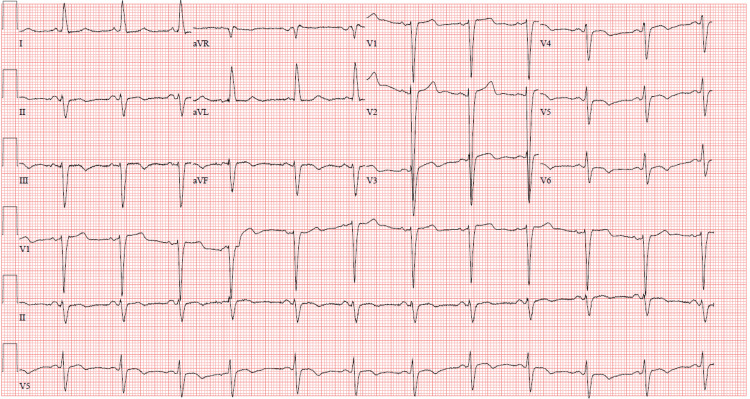
Electrocardiogram showing normal sinus rhythm, left axis deviation, and left bundle branch block aVR, augmented vector right; aVL, augmented vector left, aVF, augmented vector foot

On presentation, the vital signs were within normal limits. A neurological examination showed left homonymous hemianopia and no other cranial nerve, motor, or sensory deficits. A complete blood count and comprehensive metabolic profile were unremarkable. The electrocardiogram showed normal sinus rhythm with left axis deviation and left bundle branch block (LBBB) suggestive of left ventricular hypertrophy (Figure 1).

**Figure 2:**
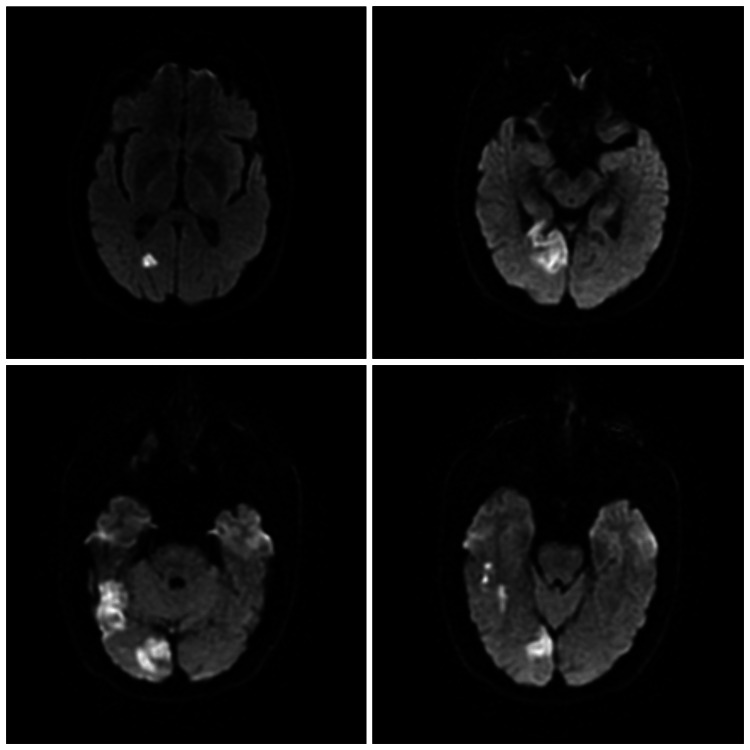
Diffusion-weighted MRI sequence showing increased signal intensity in the right occipital and right posterior temporal region MRI: magnetic resonance imaging

**Figure 3:**
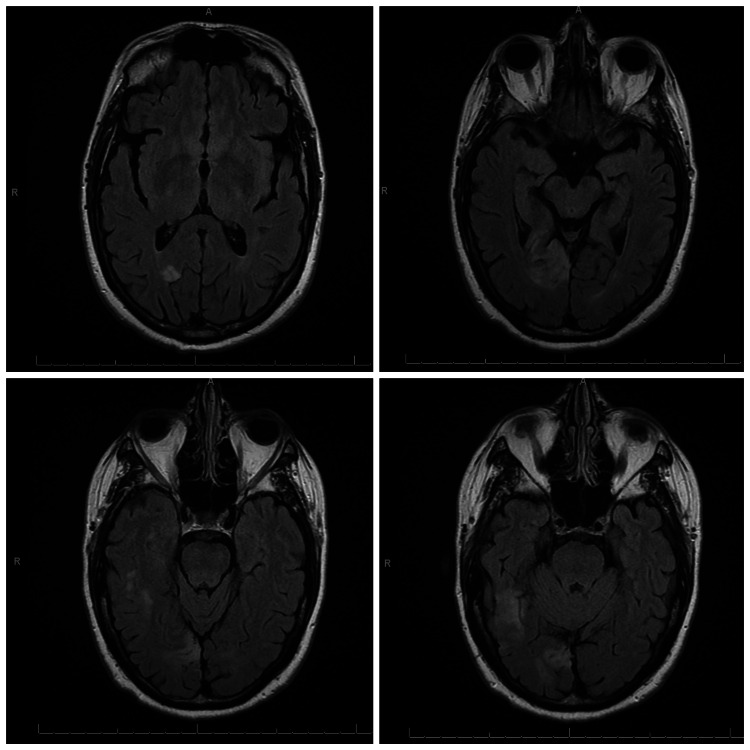
T2-weighted MRI sequence showing increased signal intensity and vasogenic edema in the right occipital lobe and right posterior temporal lobe region MRI: magnetic resonance imaging

Computed tomography (CT) scan of the head without contrast revealed an asymmetric area of low attenuation with loss of gray-white matter differentiation in the right occipital lobe, concerning acute/subacute infarction. Magnetic resonance imaging (MRI) of the brain showed marked hyperintensity and associated vasogenic edema in the right occipital lobe and right temporal region suggesting acute ischemic stroke of likely embolic origin (Figures 2-3).

The patient was diagnosed with non-compaction cardiomyopathy five years ago through cardiac magnetic resonance imaging (MRI). Since then, he is undergoing yearly follow-up multigated acquisition (MUGA) scans. The last scan 10 months ago showed a left ventricular ejection fraction (LVEF) of 55% and no intracardiac thrombus.

In this hospital course, a transesophageal echocardiogram (TEE) was performed for ischemic stroke. It showed an ejection fraction of 28%, no intracardiac thrombus, and multiple apical left ventricular trabeculations consistent with left ventricular non-compaction (Videos 1-2). He had been on cardiac rhythm monitor many times since the diagnosis and was not found to have atrial fibrillation or any other arrhythmias. Notably, the patient did not have any symptoms suggesting congestive heart failure such as dyspnea, fatigue, or fluid overload.

**Video 1:** Transgastric short-axis view on transesophageal echocardiogram (TEE) showing endocardial trabeculations

**Video 2:** Mid-esophageal long-axis view on transesophageal echocardiogram (TEE)

The patient was started on aspirin 81 mg once daily and atorvastatin 80 mg once daily for the secondary prevention of ischemic stroke. For the management of heart failure with reduced ejection fraction, goal-directed medical therapy with carvedilol and sacubitril-valsartan was initiated. Due to the discovery of low ejection fraction and thromboembolic event with non-compaction cardiomyopathy, the patient was transitioned to apixaban monotherapy with discontinuation of the low-dose aspirin.

The annual MUGA scan two months later showed improvement in the ejection fraction to 41%. The regadenoson stress test did not show any evidence of reversible or irreversible ischemia, which was done to rule out any ischemic cause of heart failure. On follow-up, he had a complete resolution of visual field deficits. He also denied shortness of breath, weight gain, leg swelling, or other symptoms suggesting congestion due to heart failure. He remains on therapeutic anticoagulation with apixaban.

## Discussion

Ischemic stroke can be caused by multiple factors such as embolism from a high-risk cardiac source. Left ventricular non-compaction (LVNC) is one of the causes that if accompanied by reduced ejection fraction leads to sluggish blood flow due to dilated and poorly contracting ventricles and increases the risk of thrombus formation in the intertrabecular recesses [6]. Along with thromboembolic events, LVNC can also cause progressive heart failure and arrhythmias [7]. There has been a debate regarding whether anticoagulation is prophylactically needed in patients with LVNC or if it alone is a risk factor for thromboembolic stroke [8]. In a retrospective study comprising LVNC patients conducted by Stöllberger et al. [7], the major cause of stroke and thromboembolism was deemed to be cardioembolic, which was mostly seen in the ones with reduced ejection fraction. Other causes of stroke identified were atherosclerosis and atrial fibrillation. The incidence of stroke in LVNC in a retrospective study of 104 patients was found to be 15% [9], and in another study with 62 patients with LVNC and 62 control patients matched with regard to age, sex, and ejection fraction, the incidence of stroke or thromboembolism was 10% in patients with LVNC and 15% in controls [6]. Hence, LVNC is concluded not to be an independent risk factor for stroke or thromboembolism, and no anticoagulation was advised in the absence of risk factors. Therapeutic anticoagulation is recommended in patients with an ejection fraction of <40%, atrial fibrillation, or a history of a thromboembolic event [7,8]. In this case, due to multiple areas of brain involvement seen on MRI, the cause of the stroke was deemed to be thromboembolism from LVNC. Hence, even though the ejection fraction improved after guideline-directed therapy, it was decided to continue him on therapeutic anticoagulation with apixaban.

The trabecular state in NCC also predisposes to conduction abnormalities and tachyarrhythmias as those trabeculations are involved in the development of the Purkinje system. In a study [10], the most common conduction abnormality found was left bundle branch block (LBBB). Other arrhythmias were life-threatening ventricular tachyarrhythmias, corrected QT (QTc) prolongation, early repolarization, paroxysmal supraventricular tachycardia (PSVT), and atrial fibrillation. Patients with LVNC who develop Wolff-Parkinson-White (WPW) syndrome, atrioventricular (AV) reentrant tachycardia, or AV reentrant nodal tachycardia may need aggressive assessment with further electrophysiologic studies and possible ablation treatment. Due to the risk of sudden cardiac death in the cohort of these patients, implantable cardioverter-defibrillators (ICDs) should also be considered in patients with LVNC who have symptomatic ventricular arrhythmias, ventricular arrhythmias refractory to ablation, or a history of an aborted cardiac arrest [11]. There is limited data regarding cardiac resynchronization therapy or biventricular pacing. Patients with heart failure in whom biventricular pacer or ICD was implanted as per guidelines have noticed an improvement in LVEF, LV end-diastolic volume, and functional capacity [12-14], with no cross-referencing data yet.

The diagnosis of LVNC is made by echocardiography and cardiac magnetic resonance imaging (MRI). Jenni et al. [15] proposed a criterion based on the end-systolic ratio of non-compacted to compacted layers of above 2. Chin et al. [1] proposed using the end-systolic ratio of the distance from the epicardial surface to the trough of trabecular recesses, to the peak of trabeculations of 0.5 or less. Stöllberger et al. [16] created a criterion agreed upon by experts in LVNC who defined LVNC as >3 prominent trabecular formations along the left ventricular endocardial border in end-diastole. To fit in the defined characteristics, trabeculations must be distinct from the papillary muscles, false tendons, or aberrant bands; should move synchronously within the myocardium; and should be the non-compacted part of a two-layered myocardial structure with apparent perfusion of the intertrabecular spaces from the ventricular cavity at end-diastole on color Doppler or contrast echocardiography.

Cardiac magnetic resonance imaging has become the method of choice to confirm or rule out left ventricular non-compaction because reliable quantitative delineation is dependent on the adequacy of acoustic windows, particularly observed during the assessment of anterior and lateral segments [17]. The criteria for diagnosis by cardiac MRI began with Petersen et al., who described the criteria as the ratio of non-compacted myocardium to compacted myocardium greater than 2.3 during the diastole (sensitivity of 86% and specificity of 99%) [18]. Later, Jacquier et al. described another method as trabeculated left ventricular mass above 20% of total mass (sensitivity of 93.7% and specificity of 93.7%) [19].

In this case, with a known diagnosis of LVNC, a low ejection fraction presumably developed as a consequence of this cardiomyopathy, which could have led to thromboembolism. As per the available literature, the patient was started on therapeutic anticoagulation with direct oral anticoagulant (DOAC). There is no data yet mentioning the superiority of one form of anticoagulant over another. A preventive strategy for the prevention of stroke in LVNC is routine cardiac imaging for the evaluation of ejection fraction and intracardiac thrombus and the initiation of therapeutic anticoagulation appropriately in conditions as described above. Also, the patients need to be cognizant of symptoms of congestive heart failure with prompt imaging to detect ejection fraction. Further case reports and outcome measurements would contribute to further understanding and characterization of the disease process.

## Conclusions

In this case report, as the patient had reduced ejection fraction and an ischemic stroke likely from a cardiogenic source, he was started on therapeutic anticoagulation. This demonstrates the possibility of rapid progression of heart failure in LVNC, which may not be detected on annual screenings. Also, not all patients with reduced ejection fraction may be symptomatic to seek evaluation and may present with a thromboembolic event as highlighted in this case.
